# Antibiotics combined with vaginal probiotics in the embryo transfer cycle of infertile patients with chronic endometritis

**DOI:** 10.3389/fcimb.2024.1494931

**Published:** 2024-11-28

**Authors:** Ping Hu, Mengyue Chen, Lu Zhu, Bing Song, Chao Wang, Xiaojin He, Guanjian Li, Yunxia Cao

**Affiliations:** ^1^ Reproductive Medicine Center, the First Affiliated Hospital of Anhui Medical University, Hefei, Anhui, China; ^2^ National Health Commission Key Laboratory of Study on Abnormal Gametes and Reproductive Tract, Hefei, Anhui, China; ^3^ Key Laboratory of Population Health Across Life Cycle, Ministry of Education of the People’s Republic of China, Hefei, Anhui, China; ^4^ Reproductive Medicine Center, Shanghai General Hospital, Shanghai Jiao Tong University School of Medicine, Shanghai, China

**Keywords:** chronic endometritis, vaginal probiotics, antibiotics, *Lactobacillus*, IVF-ET

## Abstract

**Introduction:**

Chronic endometritis (CE) is a prolonged, mild inflammation of the endometrial lining. This study investigated the impact of the impact of antibiotic treatment combined with vaginal *Lactobacillus* on pregnancy outcomes in infertile patients with CE during frozen embryo transfer (FET) cycles.

**Methods:**

A retrospective analysis was performed on the clinical data of 7,385 patients who underwent FET. After applying the inclusion and exclusion criteria, 254 patients diagnosed with CE were eligible for inclusion. Of these, 119 patients received antibiotics alone, whereas 135 were treated with a combination of doxycycline and vaginal *Lactobacillus*. All patients underwent embryo transfer within 6 months following treatment. The general characteristics and pregnancy outcomes of the first FET cycle post-treatment were compared between the two groups.

**Results:**

There were no statistically significant differences between the two groups in terms of general characteristics, clinical pregnancy rate, early miscarriage rate, and ectopic pregnancy rate. Patients who received a combination of doxycycline and *Lactobacillus* showed a higher biochemical pregnancy rate compared to those who received doxycycline alone, though this difference was not statistically significant (70.37% vs. 64.71%, P=0.313). Furthermore, the incidence rate of premature rupture of membranes was lower in the doxycycline-*Lactobacillus* group than in the doxycycline group (50.00% vs 33.33%, P=0.037).

**Conclusions:**

Although this study observed the potential benefits of the antibiotic and vaginal probiotic treatment regimen in increasing the biochemical pregnancy rate and reducing the incidence of premature rupture of membranes, the current findings are insufficient to recommend the combined use of antibiotics and vaginal *Lactobacillus* as an intervention to improve reproductive outcomes in infertile patients with CE.

## 1 Introduction

*In vitro* fertilization-embryo transfer (IVF-ET) is a pivotal methodology in assisted reproductive technologies and is widely utilized in clinical settings. Chronic endometritis (CE) is characterized by persistent inflammation of the endometrial lining, specifically marked by plasma cell infiltration into the endometrial stroma (Kitaya et al., 2018). The insidious onset of CE, coupled with its lack of pronounced systemic symptoms, often results in an overlooked clinical presentation, manifesting only as minor symptoms, such as abnormal uterine bleeding, dyspareunia, pelvic pain, and unusual vaginal discharge (Moreno et al., 2018).

The diagnosis of CE requires endometrial biopsy and the exclusion of other endometrial lesions through endometrial biopsy (Vitale et al., 2023). Hysteroscopy is the targeted biopsy method with the highest diagnostic accuracy and cost-effectiveness. Currently, the primary diagnostic criteria for CE are hysteroscopic examination and histopathological evaluation of the endometrial biopsy, with the presence of plasma cell infiltration within the endometrial stroma serving as a key diagnostic marker (Song et al., 2019). Advances in assisted reproductive technologies and the broader application of hysteroscopy have illuminated the prevalence of CE in infertile women, ranging from 10.4% to 56.8%, and in women with recurrent implantation failure from 14% to 67.5% (Liu et al., 2018; Li et al., 2023). CE is thought to impede the embryo implantation process through mechanisms involving inflammation, immune responses, and local vascular abnormalities, thereby contributing to failures in embryo transfer implantation (Vitagliano et al., 2018; Liu et al., 2022b).

The advent of next-generation microbial detection technologies has challenged the belief that the uterine environment is sterile. Emerging studies utilizing metagenomics and 16S RNA sequencing have confirmed the presence of an intrinsic microbiota within the uterine cavity (Puente et al., 2020). The uterine microbiome of healthy women predominantly consists of *Lactobacillus* species, whereas in patients with CE, a shift toward the dominance of non-*Lactobacillus* bacteria, including an increased presence of pathogenic bacteria, such as Staphylococcus, Gardnerella, Streptococcus, and Enterococcus, has been observed (Liang et al., 2023). Notably, a microbiome dominated by non-*Lactobacillus* species within the endometrium significantly correlates with reduced rates of implantation, pregnancy, ongoing pregnancy, and live births in the context of assisted reproductive treatments (Moreno et al., 2016; Gao et al., 2024; Guan et al., 2023).

The origin of the bacteria within the uterine cavity remains unclear, although it may be associated with retrograde migration from the vaginal flora through the cervical mucus. Previous studies comparing the endometrial and vaginal microbiomes of women with and without CE have highlighted a continuum of microbial populations across the female reproductive tract, with variations in the vaginal microbiome showing a clear association with CE (Lozano et al., 2021; Tanaka et al., 2022). A recent study has demonstrated a symbiotic and co-varying relationship between the vaginal and uterine microbiomes, suggesting that disturbances within the vaginal microbiota can lead to dysbiosis of uterine microecology, potentially exerting either destructive or protective effects on the endometrial tissue (Wang et al., 2021).

Frontline treatment for CE in clinical practice predominantly involves antibiotic therapy, such as oral doxycycline (Kitaya and Yasuo, 2023). Reports have indicated that a single course of antibiotic therapy yields a cure rate for CE ranging from 31.3% to 92.3% (Cicinelli et al., 2021). Data suggest that women with a history of recurrent pregnancy loss and untreated CE exhibit markedly low live birth rates (7%), which significantly improve following antibiotic treatment and the alleviation of primary inflammatory conditions (McQueen et al., 2014; Liu et al., 2022a). However, the sole use of antibiotics in the treatment of CE still faces challenges: while antibiotics inhibit pathogenic bacteria, they often also disrupt the normal genital tract microbiota and cannot address issues such as the reduction of beneficial bacteria like *Lactobacillus*. Long-term or high-dose use of antibiotics may also lead to increased bacterial resistance, a high recurrence rate of CE, and even problems such as bacterial translocation and secondary infections. Therefore, it is imperative to explore novel therapeutic strategies that not only enhance the cure rate of CE but also improve the reproductive tract microenvironment. Combining antibiotic treatment with the administration of vaginal probiotics, specifically Lactobacilli, is a promising therapeutic approach.

This study performed a retrospective analysis comparing the clinical outcomes of infertile patients with CE treated solely with antibiotics and those receiving a combination of antibiotics and vaginal *Lactobacillus* therapy in subsequent frozen embryo transfer (FET) treatments. This study aimed to identify the potential of vaginal probiotics to enhance clinical pregnancy and live birth rates in patients with CE.

## 2 Materials and methods

### 2.1 Study population

This retrospective analysis reviewed the clinical data of patients (n = 7,385) who underwent FET at the Reproductive Medicine Center of the First Affiliated Hospital of Anhui Medical University from July 2021 to August 2022. The inclusion criteria included: patients who underwent FET in our center during this period, were aged ≤ 40 years, had undergone hysteroscopy and endometrial biopsy within 6 months before embryo transfer, and were pathologically confirmed as having chronic endometritis after surgery, with a total of 343 cases. Exclusion criteria included: other types of endometrial inflammation (such as acute, subacute or tuberculous endometritis); at least three previous failed embryo transfer attempts; bacterial vaginosis; moderate-to-severe intrauterine adhesions; uterine malformations; submucosal fibroids; endometrial cancer or atypical hyperplasia; hydrosalpinx; severe male factor infertility; endometriosis or adenomyosis; history of surgery for uterine fibroids and/or endometriosis; metabolic diseases; body mass index (BMI) < 18 or > 35 kg/m^2^; autoimmune diseases; antiphospholipid syndrome; thrombotic diseases; severe thyroid disorders; chromosomal abnormalities in either partner; treatment with hormones, steroids, or antibiotics other than doxycycline during the 3 months prior to or during the study period; incomplete treatment owing to severe side effects from doxycycline or vaginal Lactobacilli; and lack of informed consent. This study was approved by the Clinical Medical Research Ethics Committee of the First Affiliated Hospital of the Anhui Medical University (No. 2023800). Written informed consent was obtained from all patients.

### 2.2 Data collection

Two authors (P.H., M.Y.C.) collected clinical data from both the electronic and paper clinical record systems. The data included the general characteristics of the patients, medical history (infertility duration, health status, medication treatment, gynecological history), indications for hysteroscopy, and results of histological and immunohistochemical examinations during hysteroscopy. Clinical outcome indicators, including endometrial thickness on the day of endometrial transformation, number of embryos transferred, number of high-quality embryos transferred, biochemical pregnancy rate, clinical pregnancy rate, early miscarriage rate, and ectopic pregnancy rate, were also collected. High-quality embryos were defined as blastocysts with a score of ≥ 3BB on day 5. Clinical pregnancy was defined as an intrauterine pregnancy sac confirmed by ultrasound; “live birth” was defined as the delivery of one or more live infants; “miscarriage” was defined as the loss of a fetus before 20 weeks of pregnancy. The following formulas were used to achieve pregnancy, ectopic pregnancy, miscarriage, and preterm birth rates: Pregnancy rate is number of pregnant patients/number of transfer cycles × 100%, ectopic pregnancy rate is number of ectopic pregnancy cycles/number of transfer cycles × 100%, miscarriage rate is number of miscarriage cycles/number of clinical pregnancy cycles × 100%, and preterm birth rate is number of preterm birth cycles/number of clinical pregnancy cycles × 100%. In cases of missing data, the two authors (P.H., M.Y.C.) completed the information by contacting the patients via telephone. The final follow-up date was February 2024.

### 2.3 Diagnosis of chronic endometritis

Hysteroscopic examinations were conducted 3–7 days after the cessation of menstruation, following the exclusion of contraindications to surgery. Comprehensive assessment of the endometrium was performed using hysteroscopy. If signs of endometritis were observed, endometrial curettage and pathological examinations were performed. The curetted endometrial tissues were washed with sterile saline and fixed in 4% paraformaldehyde. Plasma cells were identified by immunohistochemical staining with hematoxylin and eosin combined with cluster of differentiation CD-138 and CD-38. CE was diagnosed based on the presence of ≥1 plasma cell per 10 high-power fields of view in the endometrial stroma (at 400x magnification) (Hirata et al., 2021). It was independently diagnosed and cross-validated by two professional pathologists.

### 2.4 Treatment regimen

**Figure 1:**
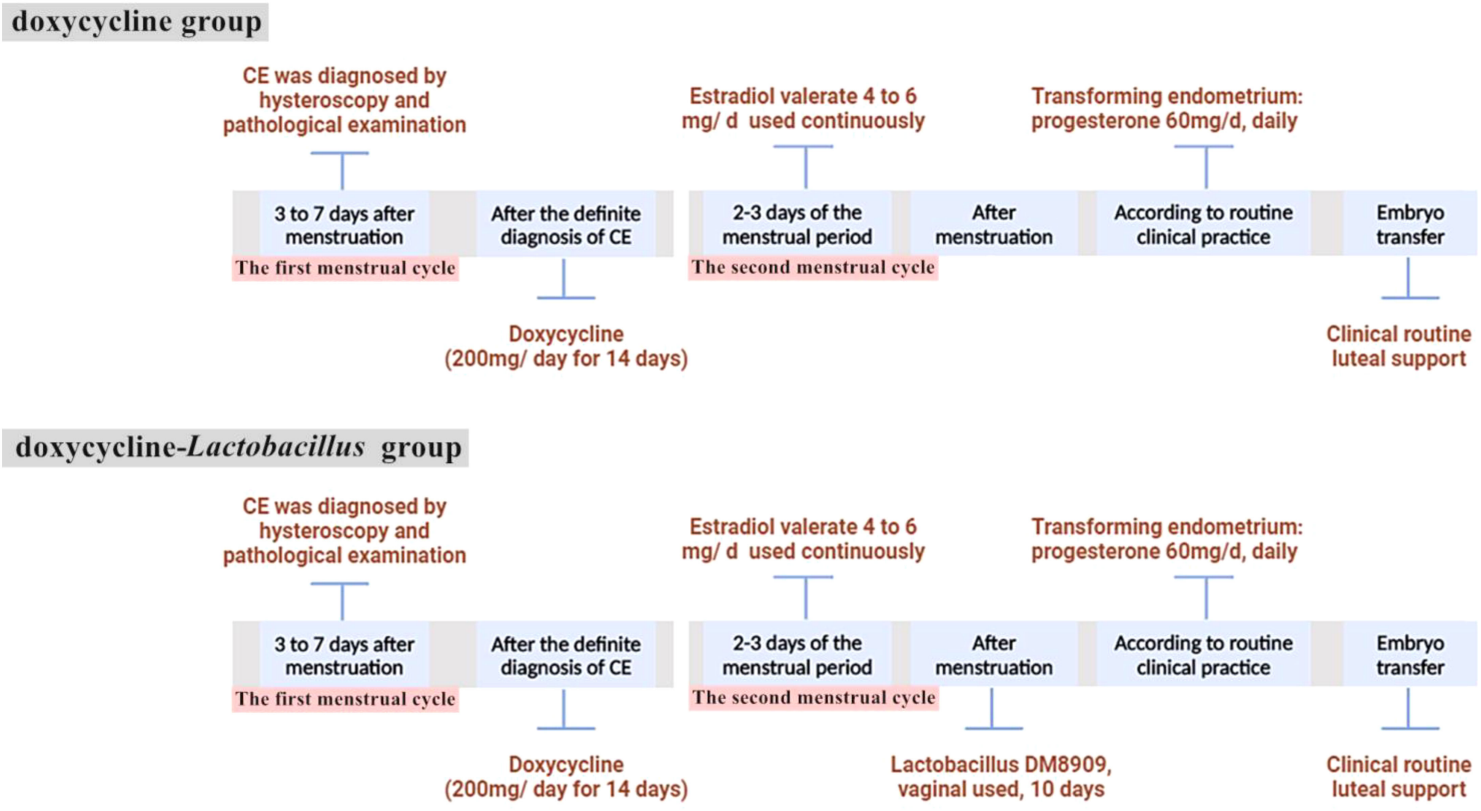
A timeline describing the treatment regimens received by the patient.

Patients in the doxycycline group commenced oral doxycycline treatment (200 mg/day for 14 days) upon diagnosis, followed by oral estradiol valerate (4 mg–6 mg per day) on the second day of the next menstrual cycle to prepare the endometrium. Patients in the doxycycline-*Lactobacillus* group also started to take oral doxycycline (200 mg/day for 14 consecutive days) after diagnosis in the first menstrual cycle, and then also took oral estradiol valerate on the second day of the next menstrual cycle. Additionally, after the menstrual period was clean, vaginal capsule containing live *Lactobacillus* delbrueckii DM8909 (trade name: Lactinex, one capsule per application, 0.25 g/capsule, each capsule contains greater than or equal to 0.25 x 1,000,000 CFU of live *Lactobacillus*, applied to the vaginal fornix at bedtime for 10 consecutive days) was added. The treatment protocol is shown in **Figure 1**.

The timing of embryo transfer was determined by the clinician based on routine clinical standards, with intramuscular progesterone (60 mg/day) administered to transform the endometrium, and blastocyst transfer was performed on the 6th day of endometrial transformation. Under ultrasound guidance, embryos were transferred to the uterus using a catheter. Biochemical pregnancy was assessed 14 days after embryo transfer by measuring serum human chorionic gonadotropin levels, and clinical pregnancy was evaluated 2 weeks later using ultrasound visualization of the gestational sac. Outcome data were collected only for the first FET cycle following antibiotic treatment.

### 2.5 Sample size calculation

According to previous research and clinical experience, the clinical pregnancy rate of patients with chronic endometritis after antibiotic treatment is approximately 40% (Kitaya et al., 2017). Assuming that the difference in clinical indicators between the two groups after treatment is 20% and anticipating a 10% loss of subjects during follow-up, 104 subjects in each group are required to study the difference in treatment effects at a 5% significance level and with 80% power (Carneiro, 2003).

### 2.6 Statistical analyses

Statistical analyses were performed using the SPSS software version 22.0. Quantitative data are presented as means ± standard deviations (x̄ ± s). Intergroup comparisons were performed using the t-test, Mann–Whitney U test, chi-squared analysis, or Fisher’s exact test, as appropriate. A P-value of < 0.05 was considered statistically significant.

## 3 Results

**Figure 2:**
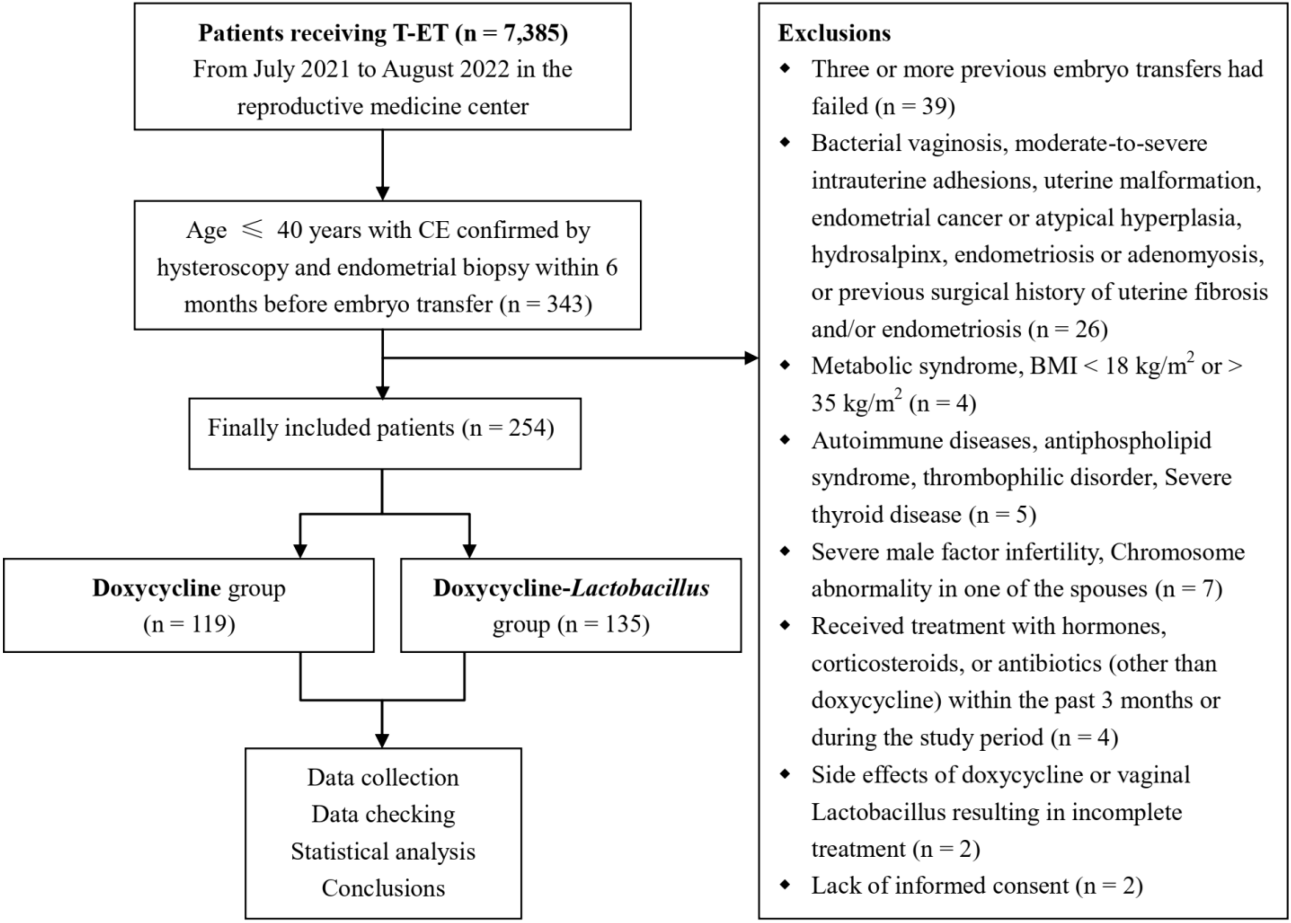
The flowchart displaying the inclusion and exclusion process in the current study.

A flowchart detailing the inclusion and exclusion criteria for the study participants is shown in **Figure 2**. A total of 254 patients who underwent FET met the inclusion criteria. Among these, 119 patients diagnosed with CE were treated solely with antibiotics (doxycycline group), whereas 135 patients received a combination of antibiotics and intravaginal *Lactobacillus* treatment (doxycycline-*Lactobacillus* group).

### 3.1 Patient profiles

The baseline characteristics of 254 patients who underwent FET are presented in **Table 1**. The cohort was entirely Asian, including 252 Chinese and 2 Vietnamese individuals. There were no statistically significant differences between the two groups in terms of age, duration of infertility, BMI, or baseline follicle-stimulating hormone levels (P > 0.05).

**Table 1:** Demographic characteristics and clinical outcomes of the study participants.

Characteristics	Doxycycline group (n = 119)	Doxycycline-*Lactobacillus* group (n = 135)	P-value
Age (years)	30.61 ± 3.90	31.00 ± 4.05	0.437
Duration of infertility	3.24 ± 2.97	3.09 ± 2.27	0.649
Body mass index (kg/m2)	22.19 ± 3.03	22.07 ± 3.22	0.761
Basal follicle-stimulating hormone	6.85 ± 2.61	7.01 ± 2.91	0.647
Endometrial thickness	10.23 ± 1.64	10.39 ± 1.40	0.402
Number of embryos transferred	1.63 ± 0.53	1.58 ± 0.50	0.460
Number of high-quality embryos transferred	1.34 ± 0.64	1.30 ± 0.56	0.600
Biochemical pregnancy rate (%)	64.71 (77/119)	70.37 (95/135)	0.313
Clinical pregnancy rate	58.82 (70/119)	59.56 (81/135)	0.907
Ectopic pregnancy rate	2.52 (3/119)	2.96 (4/135)	0.881
Early abortion rate	14.29 (10/70)	7.41 (6/81)	0.176
Live birth rate	82.86 (58/70)	77.78 (63/81)	0.419
Rates of multiple births	17.14 (12/70)	9.88 (8/81)	0.182
Premature rupture of membranes	50.00 (35/70)	33.33 (27/81)	0.037
Gestational length (days)	268.35 ± 15.81	272.75 ± 13.55	0.531

### 3.2 Clinical outcome comparison

As shown in **Table 1**, there were no significant differences between the two groups in terms of endometrial thickness, number of embryos transferred, rate of high-quality embryo transfer, clinical pregnancy rate, early miscarriage rate, and ectopic pregnancy rate (P > 0.05). Compared with the doxycycline group, the doxycycline-*Lactobacillus* group exhibited a higher rate of biochemical pregnancy, although the difference was not statistically significant (70.37% vs. 64.71%, P = 0.313). Furthermore, the incidence rate of premature rupture of membranes (PROMs) was lower in the doxycycline-*Lactobacillus* group than in the doxycycline group (50.00% vs. 33.33%, P = 0.037). During the follow-up period of all patients included in this study, hysteroscopic examination and biopsy did not reveal any severe complications or serious adverse reactions related to antibiotic treatment or *Lactobacillus* administration.

## 4 Discussion

In the current retrospective cohort study, the combination therapy of antibiotics with vaginal Lactobacilli did not enhance the clinical pregnancy or live birth rates in patients with CE undergoing FET compared with the use of antibiotics alone. Although the results indicated a slight increase in biochemical pregnancy rates in the doxycycline-*Lactobacillus* group, this difference was not statistically significant.

The endometrium is an immunological niche suitable for microbial communities, potentially playing a role in regulating inflammation and immune responses. Endometrial microbiota dysbiosis can affect reproductive outcomes during fertility treatments (Toson et al., 2022). The pathophysiological effects of endometrial microbiota on the endometrial epithelium may be related to the underlying molecular functions, cellular metabolism, genetic information, immune system, and signaling pathways (Molina et al., 2020).

The *Lactobacillus* content in the uterine cavity can affect pregnancy outcomes in women undergoing IVF. Moreno et al. found that among infertile women receiving IVF treatment, those with a *Lactobacillus*-dominant microbiota (LDM) had significantly higher rates of embryo implantation, pregnancy, ongoing pregnancy, and live birth than those with a non-*Lactobacillus*-dominant microbiota (NLDM), suggesting that the concentration of *Lactobacillus* in the uterine cavity can influence the outcomes of IVF-ET (Moreno et al., 2016). Kyono et al. reported that women with > 80% Lactobacilli in the uterine cavity had significantly higher pregnancy rates than those with < 80% Lactobacilli. Additionally, 9 of 17 women with NLDM who were treated with antibiotics in combination with oral probiotics were converted to LDM (Kyono et al., 2019). In another prospective cohort study that included 392 patients with recurrent implantation failure, 78.6% of the patients with NLDM who received vaginal probiotic suppositories plus antibiotics experienced restructuring of the endometrial microbiota composition, converting to LDM. This suggests that a combination of vaginal probiotic suppositories and antibiotics may be an effective treatment for patients with NLDM (Kadogami et al., 2020). However, owing to the small sample size and lack of representativeness in these studies and the inconsistency in the treatment methods used, it is currently challenging to form unified clinical guidelines.

The mechanisms underlying the potential role of antibiotic and vaginal *Lactobacillus* combination therapy in the treatment of CE remain unclear. Beyond enhancing the proportion of Lactobacilli within the uterine cavity, other possible mechanisms include altering the metabolic products within the vagina and uterine cavity and modifying local inflammatory responses and immune reactions within the vagina and endometrium. The *Lactobacillus* delmarkii DM8909 live bacterial capsules used in this study is mainly used for the treatment of bacterial vaginosis, and its main component is *Lactobacillus* delmarkii DM8909 live bacteria. The size of the drug is 5 capsules/box, 0.25 g/capsule, each capsule contains greater than or equal to 0.25 x 1,000,000 CFU of live *Lactobacillus*. According to the manufacturer’s instructions, this strain is isolated from the vagina of healthy women and produced by *in vitro* cultivation. The commercial strain *Lactobacillus* delbrueckii DM8909 treats bacterial vaginosis by limiting the growth, adhesion, biofilm formation, and virulence of *Gardnerella* vaginalis (Qian et al., 2021). Although the use of probiotics is temporary and Lactobacilli derived from probiotics may not necessarily colonize and proliferate within the patient’s vagina or uterus, probiotics can promote the growth of existing *Lactobacilli* or *Bifidobacteria* through their antimicrobial effects. In addition to the reduction in vaginal pH owing to lactic acid production, the antimicrobial actions of *Lactobacilli* also involve inhibition of epithelial adhesion through bacterial competition, production of bacteriocins and hydrogen peroxide to suppress microbial growth, and modulation of the local immune system (Aroutcheva et al., 2001). Future studies can clarify whether vaginal Lactobacilli can increase the quantity and proportion of Lactobacilli within the uterine cavity by examining the changes in the vaginal and uterine microbiota post-application. A comparison of the expression of local inflammatory markers, leukocyte infiltration, lipopolysaccharide content, metabolic characteristics, and changes in the uterine endothelium post-treatment can determine whether vaginal Lactobacilli can improve pregnancy outcomes by enhancing local immune responses, energy metabolism, and decidualization within the endometrium.

The correlation between the use of *Lactobacillus* spp. and the incidence of PROMs found in this study is also merits attention. Previous studies have shown a relationship between PROMs and reproductive tract microbiota, suggesting that probiotics may have a protective effect against PROMs (Bennett et al., 2020; Kavak et al., 2014). Given that PROMs may partly result from inflammatory processes at the fetal–maternal interface, probiotics, such as *Lactobacillus*, can prevent PROMs by alleviating chronic inflammation and ameliorating dysbiosis in the gut, cervix, vagina, and placenta throughout pregnancy.

This study has some limitations, including its single-center retrospective nature, limited sample size, ethnic diversity, and limitations in the current treatment protocol. Over time, accumulating experience, the evolution of treatment protocols, and advancements in embryo culture techniques may challenge the comparability of results between the groups. In the future, the efficacy of targeted modulators for the female reproductive tract microbiome, including the uterine microbiota, may depend on local factors, such as pH, temperature, fluid and secretion composition, enzymatic metabolism, clearance rates, tissue renewal during the menstrual cycle, and hormonal fluctuations. Further studies are required to assess the effects of probiotics administered at different times, types, and routes during the FET cycle on the uterine endometrial microbiome. Moreover, the patients with CE in this study were diagnosed during hysteroscopic examination before embryo transfer. Considering the absence of a history of repeated implantation failure and the time and economic costs of repeated surgical treatments, most patients did not undergo a second post-treatment endometrial examination, thus precluding a comparison of the post-treatment CE resolution rate and Lactobacilli content in the vagina and uterine cavity between the two groups. Endometrial sampling may not fully reflect the state of the entire uterine cavity and endometrium because of its reliance on limited sample collection. Therefore, different treatment outcomes may arise from varying post-treatment CE resolution rates and Lactobacilli content. Future studies should consider re-examining patients post-treatment with hysteroscopy and endometrial pathology to analyze the uterine and vaginal microbiota and CE resolution rates and determine ways to improve treatment outcomes.

## 5 Conclusion

This retrospective cohort study did not find any significant benefits of antibiotic and vaginal *Lactobacillus* combination therapy in achieving clinical pregnancy or live birth rates in patients with CE. However, the current study observed a potential effect of combined vaginal *Lactobacillus* and antibiotic administration in increasing biochemical pregnancy rates and reducing the incidence of premature rupture of membranes in patients with CE. Modulation of the reproductive tract microbiome to improve reproductive outcomes is a promising area of study, necessitating well-designed clinical and preclinical studies for further evaluation.

## Data Availability

The original contributions presented in the study are included in the article/supplementary material. Further inquiries can be directed to the corresponding author/s.
